# The Role of Anti‐Müllerian Hormone in Van Cats in Relation to Sex and Reproductive Status

**DOI:** 10.1002/vms3.70502

**Published:** 2025-07-10

**Authors:** Mehmet Yildiz, Davut Koca, Busra Nur Kilic Yildiz, Muhammed Zeyd Isik, Ali Osman Turgut, Yunus Çetin

**Affiliations:** ^1^ Department of Obstetrics and Gynecology, Faculty of Veterinary Medicine Van Yüzüncü Yil University Van Turkiye; ^2^ Department of Internal Medicine Institute of Health Sciences Burdur Mehmet Akif Ersoy University Burdur Turkiye; ^3^ Department of Internal Medicine Faculty of Veterinary Medicine Van Yüzüncü Yil University Van Turkiye; ^4^ Departmentof Animal Science Faculty of Veterinary Medicine Siirt University Siirt Turkiye; ^5^ Department of Obstetrics and Gynecology, Faculty of Veterinary Medicine Burdur Mehmet Akif Ersoy University Burdur Turkiye

**Keywords:** ELISA, hormone, intact, neutered, reproduction

## Abstract

The present study aimed to evaluate anti‐Müllerian hormone (AMH) concentrations in female and male Van cats. The study included six groups: prepubertal female cats, pubertal female cats, spayed female cats, prepubertal male cats, pubertal male cats and neutered male cats. A total of 42 Van cats were included in the study, with 7 cats in each group. Blood samples were collected from the cephalic vein of all animals, and AMH concentrations were measured from serum using the ELISA test. The highest AMH levels were observed in the prepubertal cats (*p* < 0.001). AMH concentrations were 14.33 ng/mL in prepubertal female cats and 31.91 ng/mL in prepubertal males (*p* < 0.001). In pubertal cats, AMH concentrations were 6.61 ng/mL in female cats and 10.27 ng/mL in males, with no significant difference between the sexes (*p* > 0.05). The lowest AMH levels were observed in neutered cats (*p* < 0.001). AMH concentrations were 0.109 ng/mL in spayed female cats and 0.096 ng/mL in neutered male cats, with no significant difference between the two groups (*p* > 0.05). In conclusion, the highest AMH concentrations were observed during the prepubertal period in both female cats and male cats. Furthermore, prepubertal males had significantly higher AMH levels than female cats. Measuring AMH concentrations in Van cats may help determine their reproductive status (prepubertal, pubertal or neutered).

## Graphical abstract

This study investigates the serum anti‐Müllerian hormone (AMH) concentrations in Van cats across different reproductive statuses. Blood samples were collected from prepubertal, pubertal and neutered male cats; prepubertal; pubertal as well as spayed queens. AMH levels were measured and compared to assess gonadal function and sexual development. The findings demonstrate significant differences among the groups, offering insights into the role of AMH as a potential biomarker for reproductive status in Van cats.

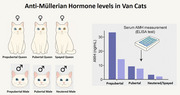

## 1 Introduction

Anti‐Müllerian hormone (AMH) is a glycoprotein hormone belonging to the transforming growth factor‐β family, produced by granulosa cells (Cate et al. 1986). Although AMH is primarily associated with foetal sex differentiation during the embryonic period, later studies have demonstrated its role in the function of both male and female reproductive organs (Teixeira et al. 2001; Josso and di Clemente 2003). AMH is exclusively expressed by gonadal somatic cells, with Sertoli cells in males (Josso and di Clemente 1999) and ovarian granulosa cells in queens being responsible for its secretion (Monniaux et al. 2012).

Unlike male foetuses, female foetuses do not produce AMH during foetal development (Themmen et al. 2016). In males, AMH secretion from Sertoli cells in the early embryonic period inhibits Müllerian duct system, preventing the formation of female reproductive structures (Rey 2005; Place et al. 2011; Akbarinejad et al. 2020). In queens, AMH production starts later, after Müllerian duct development is complete (Josso 1986). In adult queens, AMH is produced by granulosa cells, with primary follicles, secondary follicles and small antral follicles serving as the main sources of circulating AMH (Broekmans et al. 2008; Zec et al. 2011).

AMH provides highly definitive results in detecting ovarian tissue or ovarian remnants in cats with an unknown history (Alm and Holst 2018). Furthermore, the animal does not need to be in an active reproductive phase for AMH measurement. Serum AMH concentrations can be assessed even during the anestrus period, when hormone levels are at their baseline, to determine the presence of ovarian tissue (Karakas Alkan et al. 2019). In mammals, gonadectomy leads to a significant reduction in serum AMH concentrations, making AMH testing a highly specific method for differentiating between spayed and intact animals (Place et al. 2011; Claes et al. 2013; Axnér and Holst 2015). Therefore, AMH levels in female cats can be used to assess whether an individual has undergone sterilization (Axnér and Holst 2015; Heaps et al. 2017). Studies in cats have reported a statistically significant decrease in AMH levels as early as the third day after ovariohysterectomy. In neutered female cats, AMH concentrations were found to be 0.73 ng/mL on Day 3, whereas intact female cats had levels of 4.35 ng/mL (Gozer et al. 2023). Another study reported AMH concentrations below 0.04 ng/mL in blood samples collected 2 months post‐sterilization (Heaps et al. 2017). Thus, AMH concentrations can be used to make accurate assessments in spayed cats as well (Axnér and Holst 2015).

Limited information is available regarding AMH concentrations in prepubertal cats and dogs. One study in cats reported measurable AMH serum concentrations exceeding 23 ng/mL in five male and female kittens aged 4–8 weeks. Additionally, young cats have been reported to have higher AMH concentrations compared to adults (Snoeck et al. 2017; Flock et al. 2022; Ferré‐Dolcet et al. 2022). The influence of breed on AMH levels has also been documented, although studies investigating breed‐related differences in AMH concentrations remain scarce (Walter et al. 2019). A recent systematic review by Lapuente et al. (2025) emphasized the importance of AMH as a biomarker across both domestic and wild felids, highlighting its relevance in identifying reproductive status, ovarian remnant syndrome and neoplastic conditions such as granulosa cell tumours. Although studies have been conducted on AMH reference ranges in cats, no research has specifically examined AMH concentrations in Van cats. Spaying and neutering are widely practised and critical interventions for controlling the population of dogs and cats, as well as for reducing the risk of hormone‐related disorders, including mammary tumours (Howe 2006). Within this framework, AMH, a glycoprotein secreted by granulosa cells in females and Sertoli cells in males, has gained recognition as a reliable marker for evaluating reproductive status (Monniaux et al. 2012; Place et al. 2011). Although AMH has been extensively investigated in female cats, data concerning its levels in male cats remain limited, and potential breed‐specific variations have not yet been thoroughly explored (Axnér and Holst 2015; Claaßen et al. 2023). Van cats, a distinct and officially protected breed native to Türkiye, have not previously been studied in this context. Although commercially available AMH assays are frequently employed to determine spay/neuter status in both dogs and cats, reference ranges may differ depending on species, sex, age and breed. Furthermore, AMH has recently been investigated as a diagnostic marker in male cats presenting with reproductive anomalies. Posastiuc et al. (2025) reported that although AMH concentrations decreased significantly after unilateral orchiectomy in monorchid tomcats, preoperative measurements alone were insufficient to differentiate monorchidism from cryptorchidism. Currently, there are no published data establishing breed‐specific AMH reference values for Van cats.

The present study aims to determine AMH concentrations in prepubertal, pubertal and neutered or castrated Van cats in both male and female Van cats and to provide valuable reference data for accurate interpretation in clinical settings. By addressing this gap, we aim to increase the diagnostic utility of AMH testing, especially for Van cats, by complementing existing commercial tests with breed‐specific reference values.

## 2 Materials and Methods

### 2.1 Animals

This study was approved by the Van Yuzuncu Yil University Local Ethics Committee for Experimental Animals with the decision dated 30/01/2025 and numbered 2025/01‐01. The study was conducted on reproductively healthy Van cats housed at the Van Cat Research and Application Center. The necessary permissions for the study were obtained from the Van Cat Research and Application Center. A total of 42 Van cats of both sexes and various reproductive statuses were included in the study. Cats were categorized into six groups (*n* = 7 per group): prepubertal male, pubertal male, neutered male, prepubertal female cats, pubertal female cats and spayed female cats. The age of cats ranged from 6 months to 9 years, with neutered/spayed cats selected based on confirmed gonadectomy at least 1 year prior. All animals were clinically healthy and had no signs of systemic disease.

### 2.2 Blood Sample Collection and Storage

Blood samples (1–2 mL) were collected from the cephalic vein, allowed to clot at +4°C for 2–4 h, centrifuged at 3000 × *g* for 10 min, and serum samples were stored at −20°C until analysis.

### 2.3 AMH Analysis

Frozen serum samples were thawed and analysed for AMH in a single batch. Serum AMH concentrations were measured using an enzyme‐linked immunosorbent assay (AMH Gen II ELISA; Beckman Coulter) (Axnér and Holst 2015). This ELISA kit was designed with a detection limit of 0.05 ng/mL. The coefficient of variation for the assay was ≤10.3%, indicating high precision and reproducibility in measuring AMH concentrations.

Briefly, 50 µL of standards, controls and samples were first diluted and homogenized by adding 250 µL of buffer solution. Then, 120 µL of standards, controls and samples were incubated in an anti‐AMH antibody‐coated microtitration plate following the assay protocol. After incubation and washing, an antibody‐biotin conjugate was added to each well. Following a second incubation and washing step, a streptavidin–enzyme conjugate was added. After a third incubation and washing step, a chromogenic substrate was added, followed by a brief incubation before adding the stopping solution. The enzymatic conversion of the substrate was determined by measuring absorbance at a wavelength of 450 nm. Samples with results exceeding 21 ng/mL were diluted with a diluent solution, reanalysed and recalculated accordingly.

### 2.4 Statistical Analysis

Statistical analyses were performed using Minitab software. Descriptive statistics for the data were expressed as mean, standard deviation, minimum, median and maximum values. Because the data followed a normal distribution, parametric tests were used. One‐way ANOVA was employed to compare AMH levels among prepubertal, pubertal and neutered individuals within male and female cat groups separately. Independent sample *t*‐tests were used for pairwise comparisons between male and female cats. A significance level of *p* < 0.05 was considered statistically significant.

## 3 Results

Descriptive statistics for each group are presented in Table 1. In female cats, AMH ranged from 4.27 to 27.92 ng/mL (prepubertal), 4.71 to 8.78 ng/mL (pubertal) and 0.07 to 0.16 ng/mL (spayed). In male cats, AMH ranged from 21.08 to 40.51 ng/mL (prepubertal), 3.17 to 22.02 ng/mL (pubertal) and 0.08 to 0.13 ng/mL (neutered). This test exhibited 100% sensitivity and specificity.

**TABLE 1:** Anti‐Müllerian hormone (AMH) concentrations in Van cats (mean ± SEM).

Group		*N*	Mean (ng/mL)	SE mean	St Dev.	Minimum (ng/mL)	Median (ng/mL)	Maximum (ng/mL)
Female cats	Prepubertal	7	14.33	3.03	8.02	4.27	12.04	27.92
	Pubertal	7	6.61	0.68	1.79	4.71	5.97	8.78
	Spayed	7	0.11	0.01	0.03	0.07	0.11	0.16
Male cats	Prepubertal	7	31.91	2.69	7.13	21.08	30.70	40.51
	Pubertal	7	10.27	2.60	6.89	3.17	7.38	22.02
	Neutered	7	0.09	0.01	0.01	0.08	0.09	0.13

### 3.1 Female Cats

The AMH levels of female cats are presented in Table 2. The mean AMH levels in prepubertal, pubertal and spayed female cats were 14.33, 6.61 and 0.109 ng/mL, respectively. The lowest AMH concentration was observed in spayed female cats, whereas the highest AMH levels were found in prepubertal female cats. The differences in AMH levels among all female cat groups were statistically significant (*p* < 0.001).

**TABLE 2:** Anti‐Müllerian hormone (AMH) concentrations in Van cats by sex (mean ± SEM).

Group	*N*	AMH (ng/mL)		
		Female cats	Male cats	*p* value
Prepubertal	7	14.33 ± 3.03^a^	31.91 ± 2.69^a^	0.001
Pubertal	7	6.61 ± 0.67^b^	10.27±2.60^b^	>0.05
Spayed or neutered	7	0.109 ± 0.01^c^	0.096±0.01^c^	>0.05
***p* value**		<0.001	<0.001	

### 3.2 Male Cats

The AMH levels of male Van cats are presented in Table 2. The mean AMH levels in prepubertal, pubertal and neutered males were 31.91, 10.27 and 0.096 ng/mL, respectively. The lowest AMH concentration was observed in neutered males, whereas the highest AMH levels were found in prepubertal males. The differences in AMH levels among all male groups were statistically significant (*p* < 0.001).

The comparison of AMH concentrations in Van cats by sex and by period is presented in Table 2. When AMH levels were analysed by sex, the AMH concentrations in prepubertal males were found to be higher than those in prepubertal female cats (*p* < 0.001). There was no significant effect of sex on AMH levels in the pubertal and neutered groups (*p* > 0.05).

## 4 Discussion

This study is the first to investigate AMH levels in Van cats. In our study, AMH levels in all cats were detectable. As shown in previous studies, AMH analysis has 100% sensitivity and specificity in determining sterilization status, which was confirmed in both female and male cats (Axnér and Holst 2015; Place et al. 2011). Furthermore, in Van cats, the accuracy and reliability of the AMH Gen II ELISA allow the use of cat serum samples for determining AMH concentrations.

Previous studies have shown that younger cats, especially prepubertal cats, have higher plasma AMH concentrations compared to older cats. These studies have mostly been conducted on female cats (Axnér and Holst 2015; Ferré‐Dolcet et al. 2022; Flock et al. 2022; Lapuente et al. 2023). A similar pattern is observed in horses (Claes et al. 2015), female mice (Kevenaar et al. 2006) and dogs (Hill et al. 2018; Hollinshead et al. 2017). In our literature review, no study has directly compared AMH levels between prepubertal or juvenile male cats and adult male cats. In a study by Axnér and Holst (2015), the highest AMH levels were reported in prepubertal female and male cats (Axnér and Holst 2015). In a study by Snoeck et al. (2017), serum AMH levels in peripubertal cats (3–12 months) were found to be higher (9.27 mcg/L) than in pubertal cats (>12 months) (4.13 mcg/L). AMH is reported to be higher in peripubertal cats (Snoeck et al. 2017). In male humans, a decrease in AMH is observed during puberty (Lee et al. 1996). However, it has been noted that domestic cats show comparably high serum AMH concentrations with large individual variations (Place et al. 2011; Monniaux et al. 2012). Another study involving 93 female cats of different breeds and ages reported the highest AMH concentration in a 4‐month‐old female cat (Claaßen et al. 2023). The current study supports previous findings (Axnér and Holst 2015; Ferré‐Dolcet et al. 2022; Flock et al. 2022; Claaßen et al. 2023; Lapuente et al. 2023), as prepubertal female and male cats had significantly higher AMH levels compared to healthy female and male cats (*p* < 0.001). Furthermore, prepubertal male cats were found to have significantly higher AMH levels than prepubertal female cats (*p* < 0.001).

As demonstrated in other species and cats, serum AMH concentration can vary significantly among individuals. These variations are higher in healthy females and healthy males (Place et al. 2011; Monniaux et al. 2012). Many studies report that healthy male cats have higher AMH concentrations compared to intact female cats (Axnér and Holst 2015; Claaßen et al. 2023). In the current study, AMH concentrations were higher in healthy males compared to intact females. In other species, low AMH concentrations indicate ovarian ageing, whereas high AMH concentrations reflect the size of the preantral and small antral follicle pool (Monniaux et al. 2012). There is insufficient research to determine whether AMH can be used as a marker for fertility or ovarian ageing in cats. However, a recent systematic review by Lapuente et al. (2025) offers a comprehensive overview of AMH dynamics in felids, emphasizing its growing importance in clinical applications. Their results underscore the utility of AMH in diagnosing ovarian remnant syndrome, identifying granulosa cell tumours and potentially informing reproductive management in both domestic and wild cats. Nevertheless, it is important to note that the study did not include reproductively intact older individuals, which may have provided valuable information regarding age‐related variations in AMH levels. This limitation was primarily due to the unavailability of such animals within controlled clinical settings during the study period. Studies have reported that AMH concentrations in intact female cats range from 1.3 to 19 ng/mL and male cats range from 4.8 to 81.3 ng/mL (Axnér and Holst 2015). Another study involving 93 female cats of different breeds and ages reported that AMH ranged from 1.3 to 21.7 ng/mL, with an average concentration of 6.8 ± 0.5 ng/mL (Claaßen et al. 2023). In the current study, AMH concentrations in pubertal female cats ranged from 4.71 to 8.78 and in male cats from 3.17 to 22.02. Although there was a proportional increase between healthy male and female Van cats, there was no statistically significant difference (*p* > 0.05). The average AMH concentrations in intact female and male cats were 6.605 and 10.27, respectively, yielding results consistent with those reported in different cat breeds (Axnér and Holst 2015; Claaßen et al. 2023).

Because the only source of circulating AMH is the ovary (Place et al. 2011), AMH measurements are used to gather information about the presence of ovarian tissue in pets, particularly those with unknown medical histories (Alm and Holst 2018). In female cats and dogs, AMH levels can be used to determine whether sterilization surgery has been performed, regardless of the stage of the sexual cycle (Walter et al. 2019). Studies on female cats report that AMH levels decrease significantly by the third day post‐surgery. In spayed cats, AMH levels were found to be 0.73 ng/mL, whereas in intact cats, they were 4.35 ng/mL (Gozer et al. 2023). In another study, AMH concentrations in blood samples taken from spayed cats 2 months after surgery were found to be <0.04 ng/mL (Heaps et al. 2017). AMH concentrations in neutered cats can be used for accurate predictions (Axnér and Holst 2015). It has been reported that AMH concentrations in spayed cats are <0.14 ng/mL, whereas in non‐neutered cats, they range from 1.3 to 19 ng/mL (Axnér and Holst 2015). In the present study, the AMH concentrations in neutered female cats averaged 0.10 ng/mL and in male cats 0.09 ng/mL. There was no statistical difference between spayed female and neutered male cats (*p* > 0.05). Although the ovaries are the sole known source of AMH in female cats, minimal levels may occasionally be detected after gonadectomy. This could be due to the assay's high sensitivity or other yet undefined biological or technical factors. Further studies are needed to clarify this finding. The AMH concentration in intact female cats averaged 6.605 ng/mL and in males, 10.27 ng/mL. There was no statistical difference between intact female and male cats (*p* > 0.05). However, AMH concentrations in neutered male and spayed female cats were significantly lower compared to intact male and female cats (*p* < 0.001). These results are consistent with previously reported findings (Axnér and Holst 2015; Heaps et al. 2017; Gozer et al. 2023). Additionally, AMH has been investigated as a useful biomarker in diagnosing male reproductive disorders. In a study by Posastiuc et al. (2025), AMH levels were measured in tomcats suspected of monorchidism, and a significant decline in hormone levels was observed following orchiectomy. However, the researchers found that preoperative AMH concentrations were not sufficient to reliably distinguish between monorchid and cryptorchid animals. These findings highlight the utility of AMH in confirming the presence of functional testicular tissue, while also emphasizing that hormonal assessment should be complemented with imaging techniques or surgical exploration when diagnostic uncertainty exists. The dynamic changes in AMH concentrations observed across reproductive stages are likely to reflect underlying physiological regulation. In prepubertal animals, elevated AMH levels may be attributed to increased activity of Sertoli cells in males and granulosa cells in females (Josso and di Clemente 2003; Monniaux et al. 2012). As animals reach sexual maturity, hormonal feedback mechanisms such as increased inhibin B secretion and follicular development may contribute to a gradual decrease in AMH production (Kevenaar et al. 2006; Broekmans et al. 2008). Following gonadectomy, the removal of gonadal tissue leads to a marked reduction in circulating AMH, supporting its use as a potential biomarker for reproductive status (Place et al. 2011; Axnér and Holst 2015). Therefore, similar to other breeds, AMH levels in Van cats can be used to determine whether the cat is sterilized and non‐sterilized.

## 5 Conclusion

In conclusion, the highest AMH concentrations in both female and male cats were observed during the prepubertal period. Moreover, pre‐pubertal male cats have the highest AMH levels. By measuring AMH concentrations in Van cats, it is possible to determine whether the cats are prepubertal, pubertal or neutered.

## Author Contributions

**Mehmet Yildiz**: conceptualization, data curation, investigation, methodology, software, validation; visualization, writing‐original draft, writing – review and editing, supervision. **Davut Koca**: conceptualization, funding acquisition, investigation, methodology, project administration; resources; writing – original draft, writing – review and editing, supervision. **Busra Nur Kilic Yildiz, Muhammed Zeyd Isik, Ali Osman Turgut** and **Yunus Çetin**: software, visualization, writing – original draft, writing – review and editing. All authors also reviewed and approved final version of manuscript.

## Ethics Statement

This study was approved by the Van Yuzuncu Yil University Local Ethics Committee for Experimental Animals with the decision dated 30/01/2025 and numbered 2025/01‐01.

## Conflicts of Interest

The authors declare no conflicts of interest.

## Data Availability

The data that support the findings of this study are available on request from the corresponding author.
